# Supplementary material for: Allele-specific assembly of a eukaryotic genome corrects apparent frameshifts and reveals a lack of nonsense-mediated mRNA decay
Source: NAR Genom Bioinform. 2021 Sep 16;3(3):lqab082. doi: 10.1093/nargab/lqab082 (PMC8445201; doi:10.1093/nargab/lqab082)
Supplement: lqab082_Supplemental_Files [file lqab082_supplemental_files.zip › SupplementaryFigures_S1-S10.pdf]

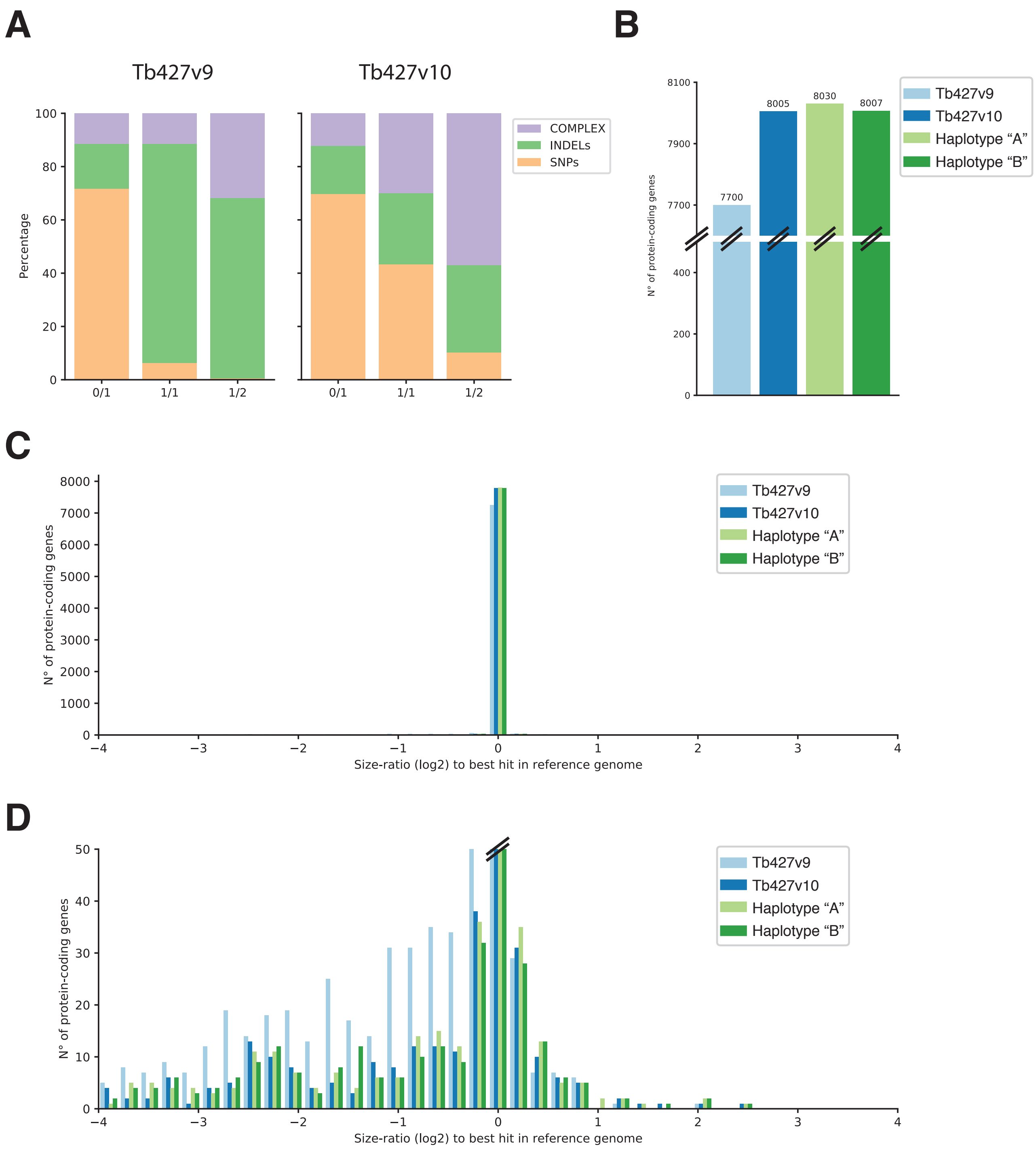

Figure S1. Variant proportion and assessment of protein content. A) Proportion of SNPs, INDELs and Complex (i.e. combination between SNPs and INDELs) variants for the different genotype scenarios (Ref/Alt1 (0/1), Alt1/Alt1 (1/1), Alt1/Alt2 (1/2)). B) Protein-coding genes annotated for the different *T. brucei* Lister 427 genome assemblies (Tb427v9, Tb427v10, haplotype “A” and haplotype “B”). C) Histogram of size-ratio (log2 scale) of protein-coding genes to the best hit in *T. brucei* TREU927 genome for the different *T. brucei* Lister 427 genome assemblies. D) Zoom-in to the plot in C).

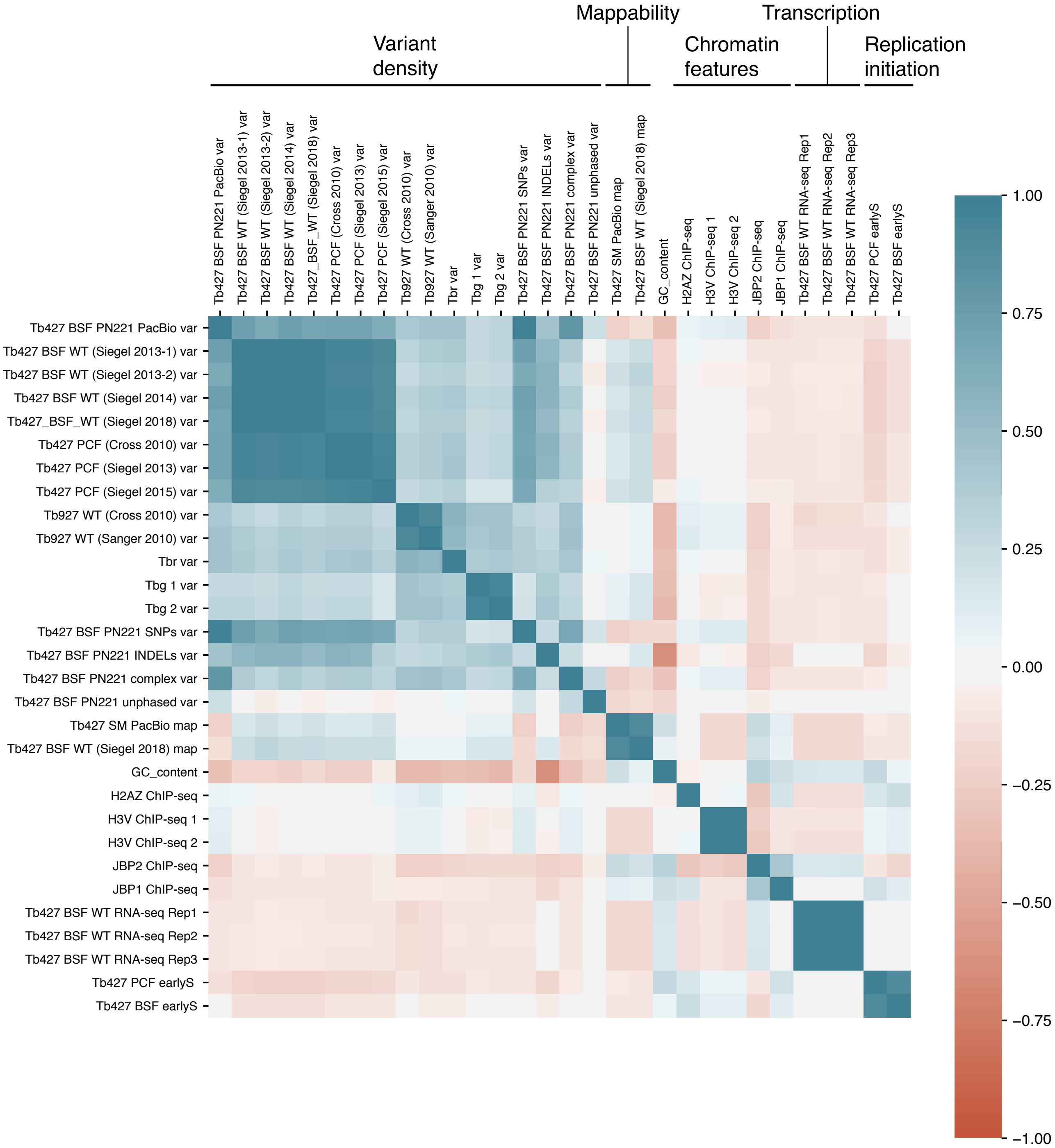

Figure S2. Correlation between variant density and different genomic features. Heatmap showing all vs all Pearson correlation for the variant density distribution of different *T. brucei* clones l, mappability, GC-content, enrichment of different chromatin factors, transcript levels and replication initiation. The description of the sequencing datasets used in this analysis, and their original publication, is listed in **Sup. Table S1**.

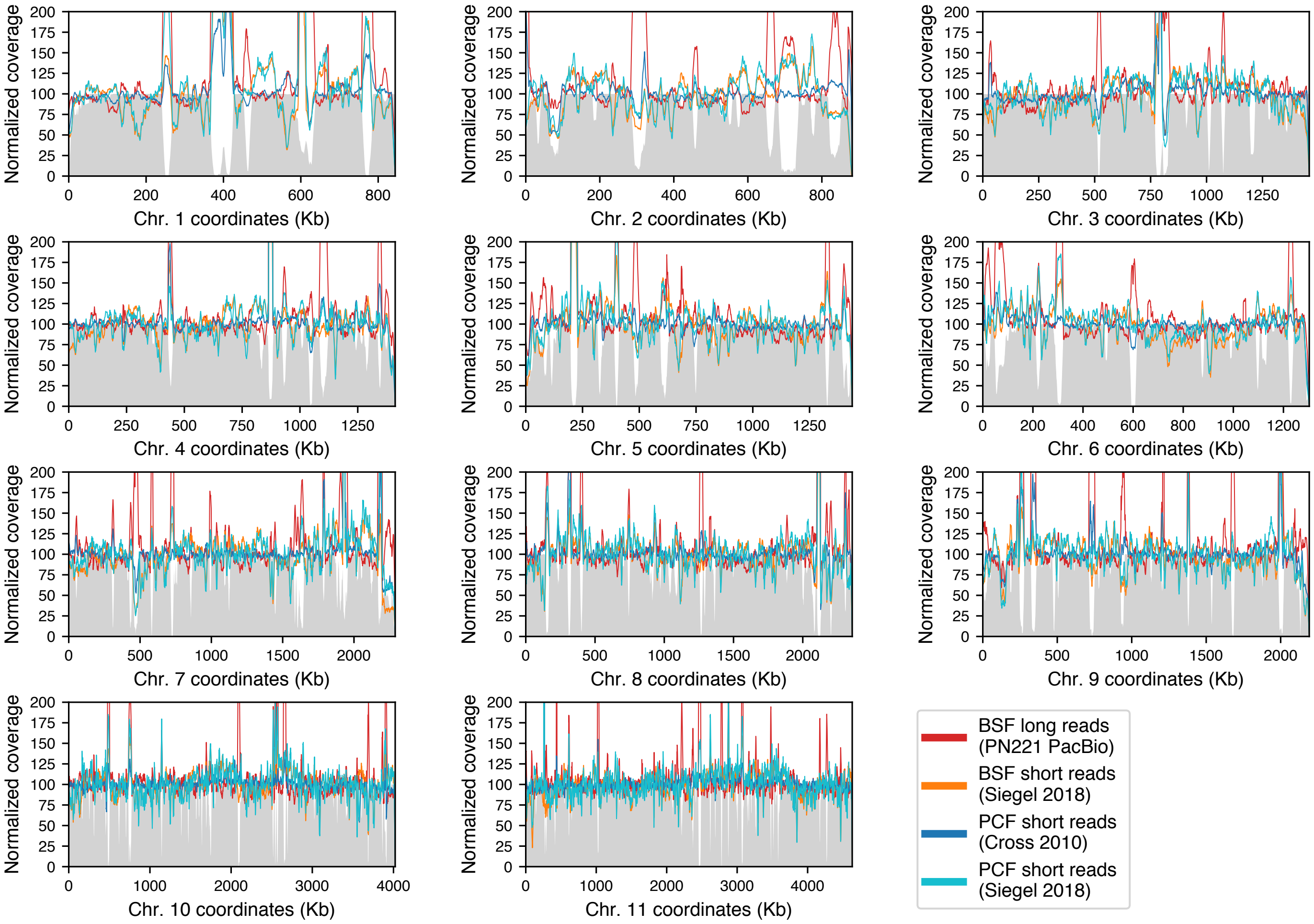

Figure S3. Genome-wide DNA-seq coverage in selected *T. brucei* Lister 427 clones. Normalized coverage along the eleven chromosomes for the DNA-seq datasets used to calculate variant density in Fig. 3. Mappability (in range 0-100) is shown as a grey filled-line.

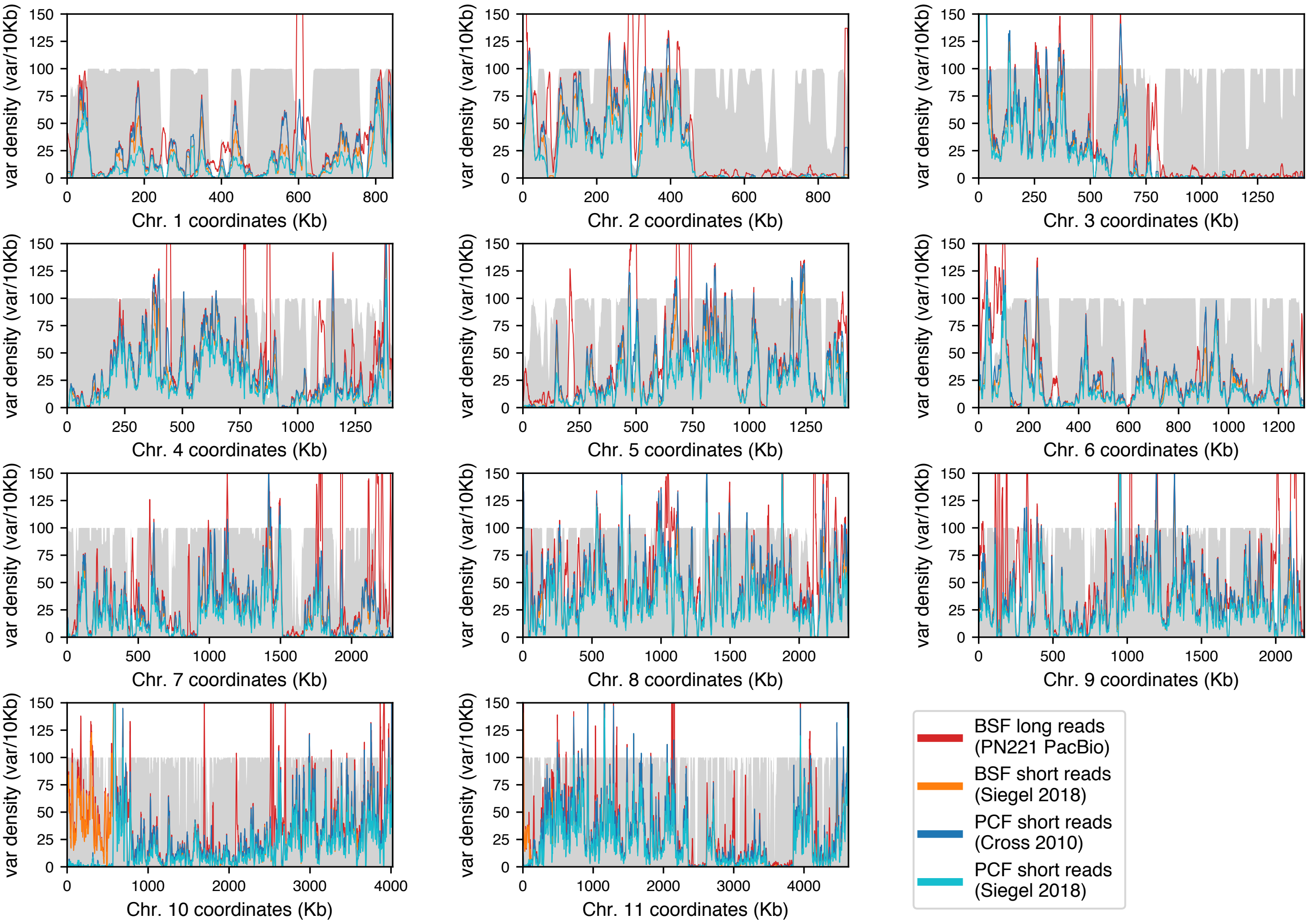

Figure S4. Genome-wide variant density in selected *T. brucei* Lister 427 clones. Variant density along the eleven chromosomes for the same DNA-seq dasets for which selected chromosomal regions were shown in Fig. 3. Mappability (in range 0-100) is shown as a grey filled-line.

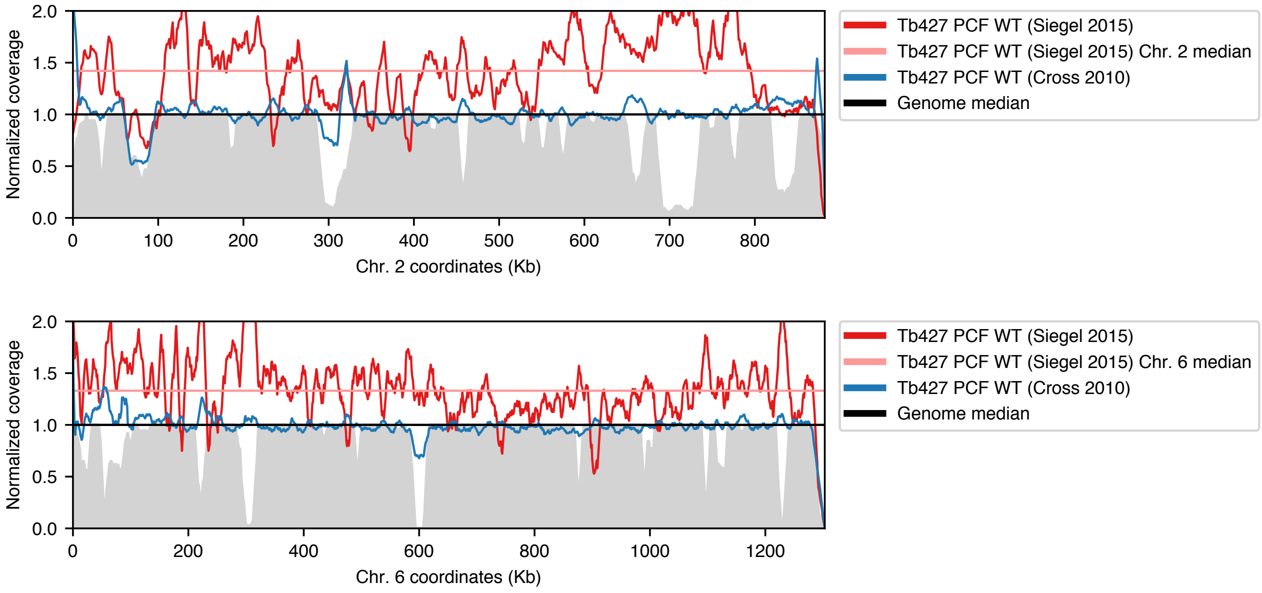

Figure S5. DNA-seq coverage in trisomic chromosomes in Tb427 PCF WT (Siegel 2015) clone. Normalized coverage density in chr 2 (top panel) and chr 6 (bottom panel) for Tb427 PCF WT (Siegel 2015) clone (dark red line) and its median (light red line), compared to Tb427 PCF WT (Cross 2010) clone (dark blue line). The genome median is set to 1 (straight black line). Mappability (in range 0-1) is shown as a grey filled-line.

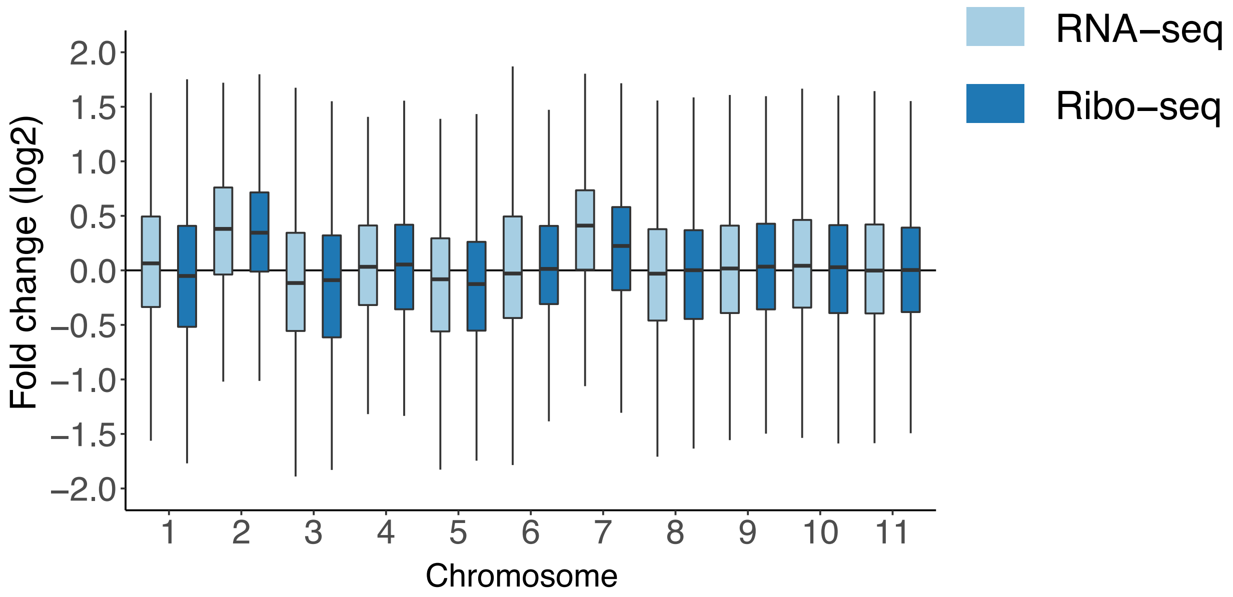

Figure S6. Genome-wide differential RNA-seq and Ribo-seq between aneuploid and diploid *T. brucei* clones. Log2-FoldChange RNA-seq (light blue) and Ribo-seq (dark-blue), pooled by chromosome, from a *T. brucei* Lister 427 PCF clone triploid for chr 2 and chr 7 over a diploid *T. brucei* TREU927 clone.

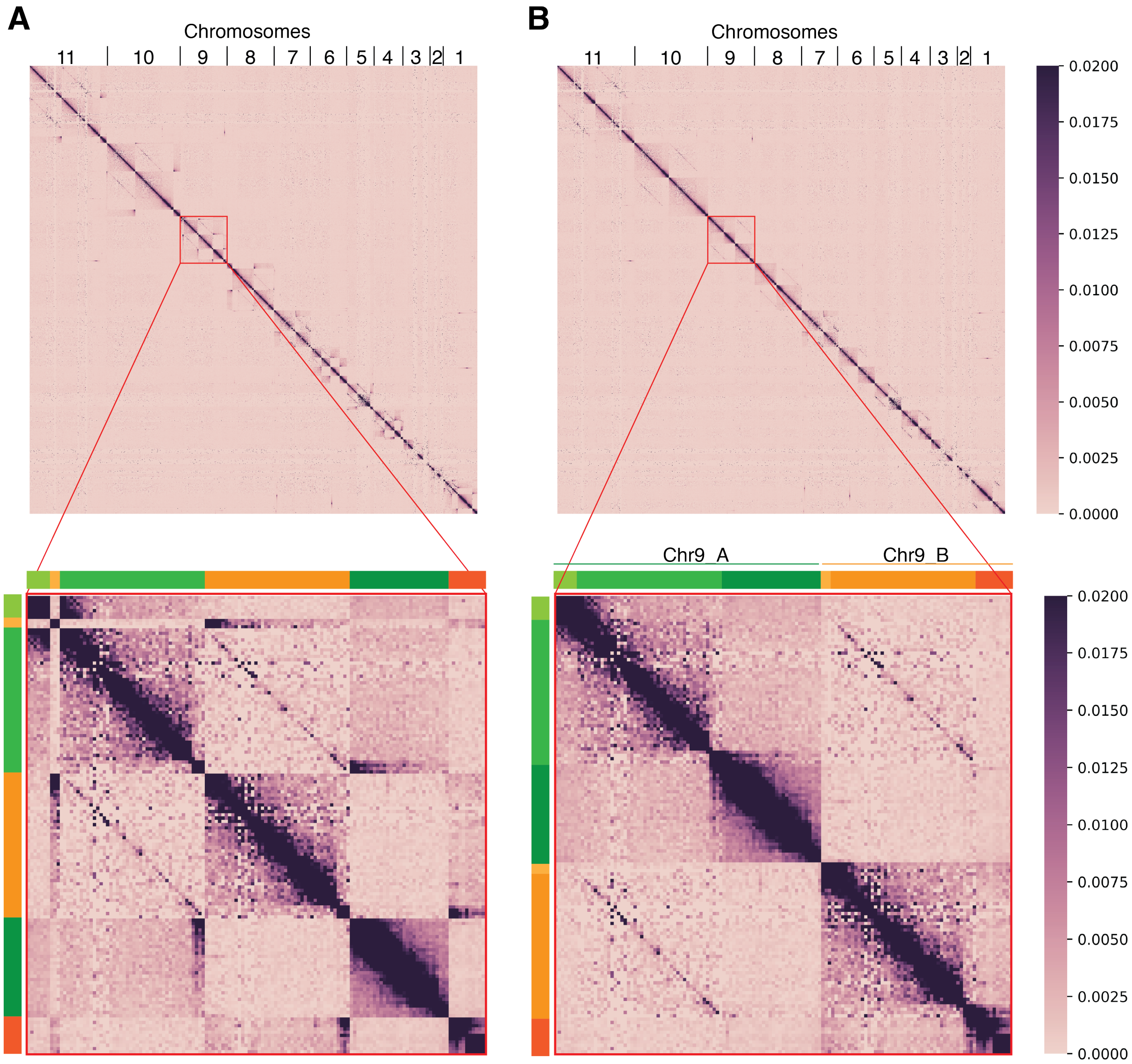

Figure S7. Hi-C interaction data between haploid-like subtelomers and phased-core alleles enables scaffolding of full homologous chromosomes. A) Hi-C interaction heatmap with the phased-core alleles and the haploid-like subtelomeres before scaffolding. Lower panel shows a zoom to the interactions between chr 9 phased-cores and haploid-like subtelomeres. B) The same as A) but after scaffolding the haploid-like subtelomeres to their interacting core allele.

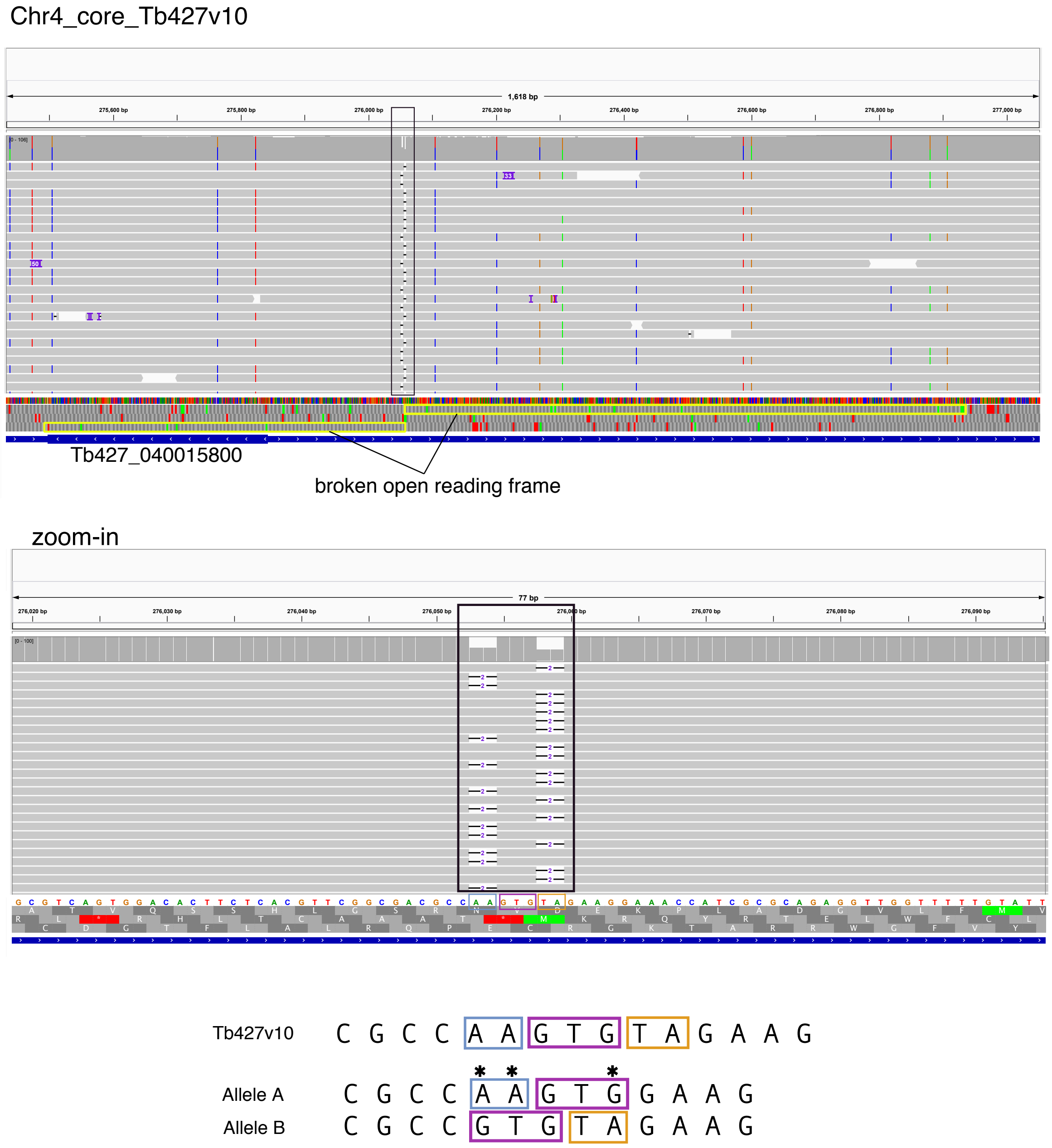

Figure S8. INDEL errors caused by nearby SNPs generating shifting sequence homology are fixed after allele-phasing. short error-corrected PacBio reads mapped to the collapsed Tb427v10 genome assembly. The upper panel is an Integrative Genome Viewer (IGV) screenshot showing a region from chr 4 containing the broken gene Tb427_040015800. Broken open reading frames are marked in yellow boxes in the translation panel. The conflicting region is marked with a black box. The lower IGV panel shows a zoom in to the conflicting region showing apparent deletions in different places for both haplotypes. At the bottom, the sequence present in Tb427v10 is shown, as well as the sequence in haplotype "A" and haplotype "B", with violet boxes showing the shifted sequence homology between the alleles generated by the several heterozygote variants (marked with asterisks).

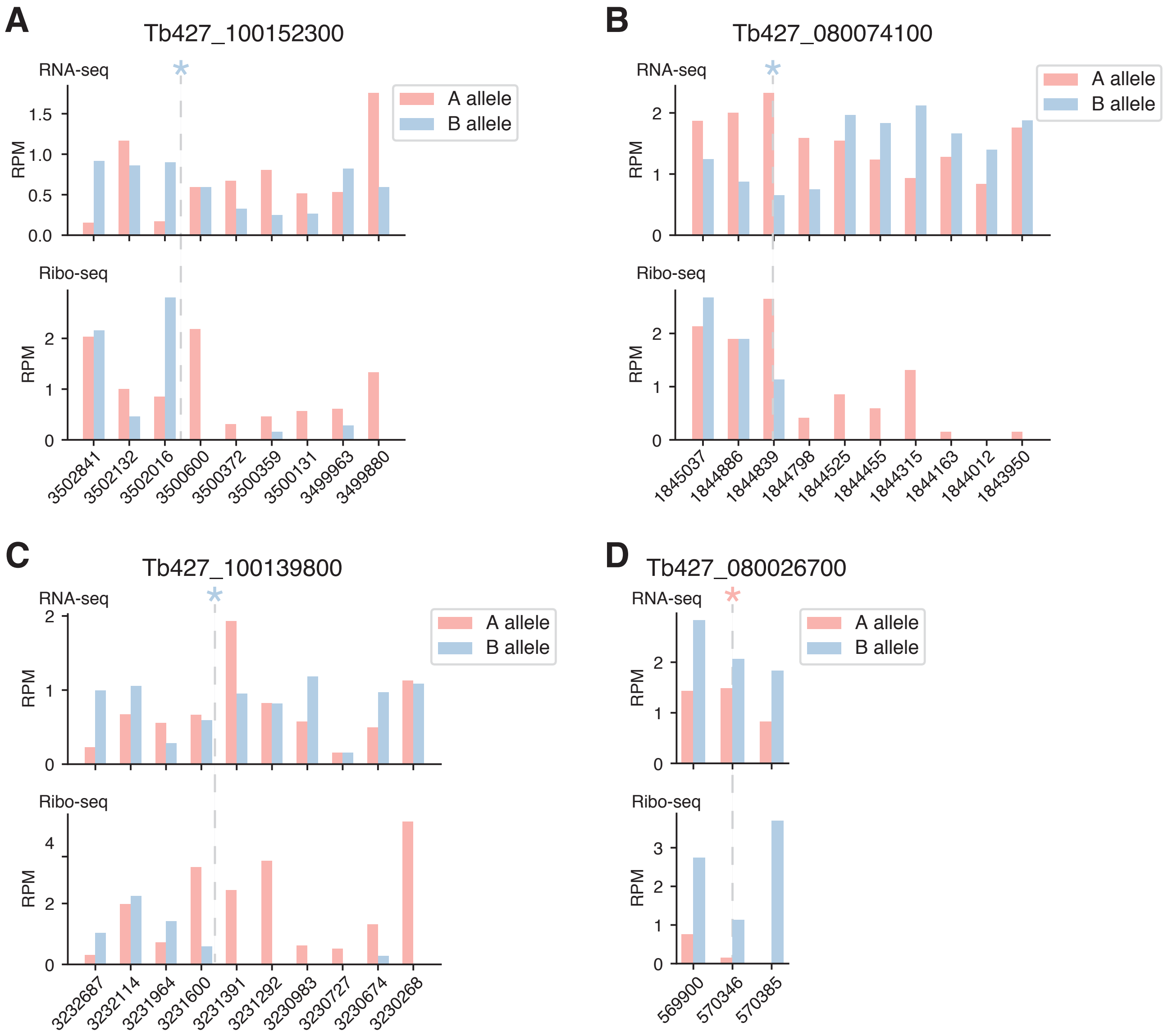

Figure S9. Allele-specific transcript and translation levels in genes with allele-specific premature termination codons (Analyses based on data published by Jensen et. al, BMC Genomics, 2014). A-D) Examples of genes with allele-specific variants leading to a premature termination codon. The geneID of the genes in the Tb427v10 genome assembly is shown on top. Below, the barplots show RNA-seq and Ribo-seq reads per million (RPM) counts for both alleles in each of the variant position (chromosomal coordinates are indicated in the bottom label). An asterisk (and a grey dashed vertical line) indicates the position of the premature termination codon and the color of the asterisk indicates which allele has it.

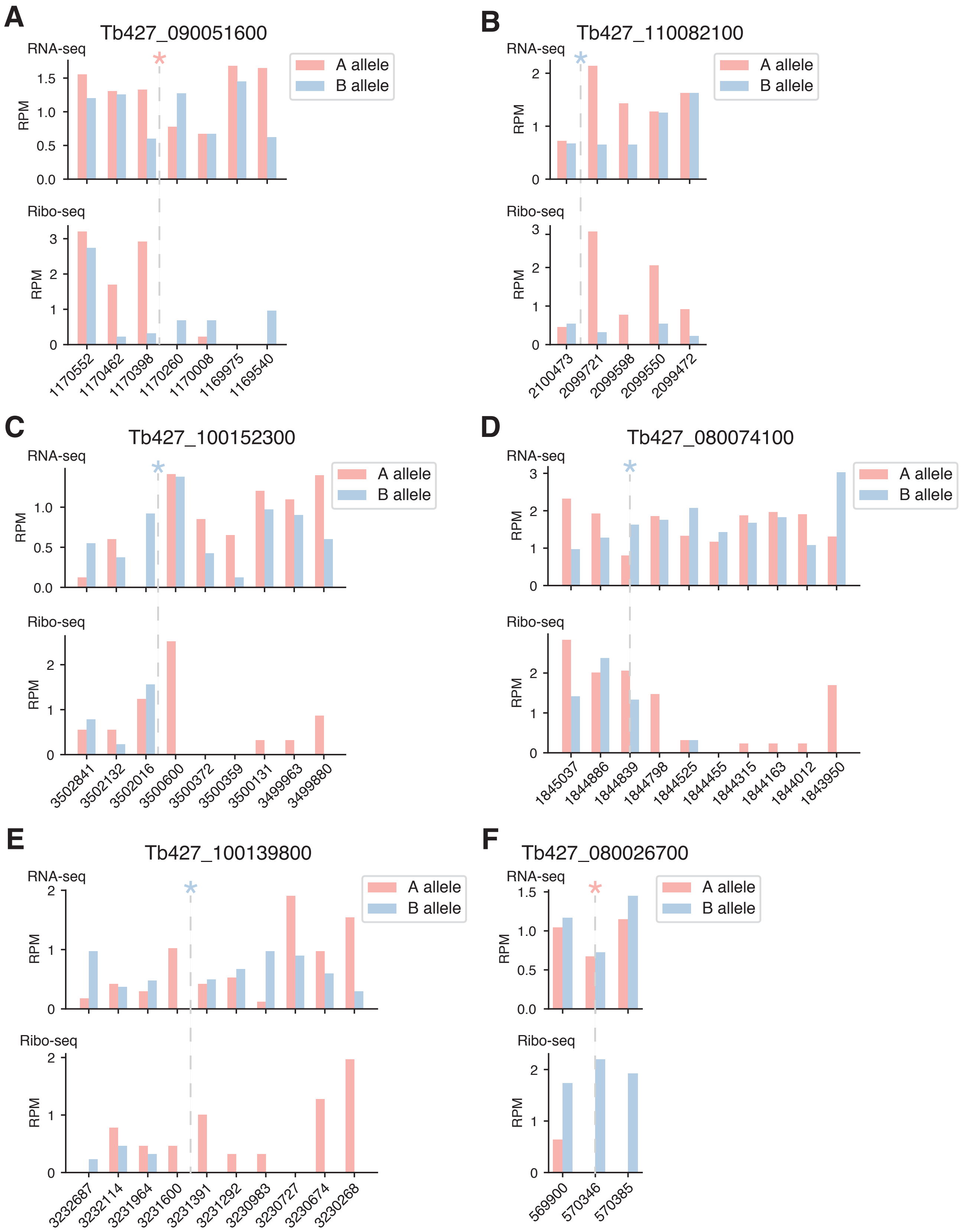

Figure S10. Allele-specific transcript and translation levels in genes with allele-specific premature termination codons (Analyses based on data published by Antwi et. al, BMC Genomics, 2016). A-F) Examples of genes with allele-specific variants leading to a premature termination codon. The geneID of the genes in the Tb427v10 genome assembly is shown on top. Below, the barplots show RNA-seq and Ribo-seq reads per million (RPM) counts for both alleles in each of the variant position (chromosomal coordinates are indicated in the bottom label). An asterisk (and a grey dashed vertical line) indicates the position of the premature termination codon and the color of the asterisk indicates which allele has it.
